# Physiological Monitoring Applications of Wearable Multimodal Fusion Systems Based on ECG and PPG: A Comprehensive Review

**DOI:** 10.3390/s26113477

**Published:** 2026-06-01

**Authors:** Chamod Rathnayake, Wenjing Chen, Sahan Jayawickrama, Dakun Lai

**Affiliations:** 1 School of Electronic Science and Engineering, University of Electronic Science and Technology of China, Chengdu 611731, China; 202424020102@std.uestc.edu.cn; 2 West China Hospital, Sichuan University, Chengdu 610041, China; chenwenjing0626@scu.edu.cn; 3 School of Mechanical and Electrical Engineering, University of Electronic Science and Technology of China, Chengdu 611731, China; sahandiwantha@gmail.com

**Keywords:** electrocardiography (ECG), multimodal signal integration, photoplethysmography (PPG), wearable technology

## Abstract

Wearable technology has become popular today not only for clinical uses but also among the general public for everyday activities such as fitness tracking and activity recognition. It’s no secret that wearable devices need to be more comfortable and that there is increasing attention to their reliability and accuracy. Therefore, researchers are making various attempts to improve these specifications. Recent research has employed multimodal fusion, rather than relying on results from a single source, such as electrocardiography (ECG) or photoplethysmography (PPG), because it enables a more comprehensive understanding of physical conditions. There are very few extensive reviews of ECG and PPG wearable multimodal fusion application systems, and they are limited to a single practical use. However, this review fills that gap and provides a comprehensive overview of recent wearable physiological monitoring technologies based on ECG and PPG, along with their modern multimodal fusion applications in the medical field. These include cuffless blood pressure estimation, stress monitoring, heart rate and heart rate variability monitoring. This presents the theoretical background, including the characteristics of ECG and PPG signals, recently developed wearable monitoring techniques for ECG and PPG, and the advantages and disadvantages of multimodal fusion. It also provides a comprehensive and comparative analysis of recent studies employing modern multimodal fusion approaches in the aforementioned medical field, as well as a discussion of the limitations and challenges of ECG-PPG wearable multimodal fusion systems reported in the literature. Therefore, this review will enable researchers to gain a complete and comprehensive understanding of the development of wearable multimodal fusion applications based on ECG and PPG.

## 1. Introduction

Physiological signals, which include electrical, biochemical, and mechanical signals, are produced by the body’s internal systems and serve as a valuable biological data source. They provide essential information for clinical applications and medical research, aiding in disease diagnosis, and play a crucial role in rehabilitation and treatment. Electrocardiogram, photoplethysmography, electromyography (EMG), electrooculography (EOG), electroencephalogram (EEG), and seismocardiography (SCG) are some examples of physiological signals, which are widely used in the fields of fitness, healthcare, and wearable technology [1,2,3,4,5]. Wearable technologies are used to collect these signals and have made tremendous progress in recent years. Companies like Apple, Fitbit, and Garmin, in particular, are successfully addressing the growing demand for wearable devices with robust hardware and software. With this demand, the “wearable electronics market” is predicted to grow from $121.7 billion to $392.4 billion in market capitalization by 2030 [6,7,8,9].

In this context, considerable attention has been devoted to the design of wearable devices based on ECG and PPG due to their established clinical relevance and ease of acquisition. A non-invasive recording of the electrical activity of the heart over time is called an ECG, which is important in assessing cardiovascular health and gaining insight into potential abnormalities [10,11]. PPG is a low-cost and non-invasive optical technique that can continuously and accurately monitor heart rate in response to changes in blood volume [12,13]. Wearable devices such as smartwatches, fitness trackers, or clothing with electrodes on the chest, wrist, or arm are used to record the ECG signal. In contrast, wearable devices such as fitness trackers and smartwatches with a light source and a photodetector placed on the wrist, earlobe, fingertip, or any other part of the body with good blood flow are used to record the PPG signal [10,12,13]. In general, these ECG- and PPG-based wearable devices have advantages such as continuous monitoring, early disease detection, remote health monitoring, non-invasiveness, and comfort, but also disadvantages such as the presence of signal artifacts and noise, limited accuracy, and limited medical validation [10,11,14,15].

To address these limitations, researchers have concentrated on creating practical applications that gather information from multiple sensors or use data fusion with higher accuracy and specificity than relying on a single sensor source, such as ECG or PPG [16]. Although several studies have investigated ECG- or PPG-based wearable systems independently, comprehensive reviews focusing on their integrated multimodal fusion applications across multiple clinical domains remain limited. To address this gap, the present review provides a comprehensive analysis of ECG-PPG wearable multimodal fusion technologies, their key physiological applications, and associated challenges. Wearable multimodal fusion devices built based on ECG and PPG can be easily integrated with machine learning (ML) and have great potential for easy real-time use in many e-health applications such as blood pressure (BP) estimation, stress detection, heart rate (HR) and heart rate variability (HRV) monitoring [16,17,18]. Fusion can be seen as being performed for these applications after feature extraction or after the final decision [16]. However, when designing multimodal fusion systems, researchers have had to pay more attention to practical issues inherent in wearable devices, such as signal synchronization, power limitations, and noise sensitivity, which have both challenged and negatively impacted the design of more reliable and successful multimodal systems [16,19]. The contributions and novelty of this review are focused on the analyses of ECG-PPG multimodal fusion of physiological monitoring applications. Furthermore, this review provides a comprehensive comparison of fusion strategies, an in-depth discussion of synchronization challenges, application-driven structured analysis, and identification of technical bottlenecks and research gaps.

This comprehensive review aims to cover recent wearable monitoring technologies based on ECG and PPG and their modern multimodal fusion applications in the medical field. Section 2 of this review provides theoretical background information along with an in-depth explanation of the characteristics of ECG and PPG signals. The next section describes the recent wearable monitoring techniques used for ECG and PPG signal detection and their pros and cons in detail. Section 4 of this review discusses signal synchronization and preprocessing techniques for wearable ECG-PPG multimodal fusion systems, which play a critical role in fusion performance. Section 5 focuses on specific key applications of the multimodal fusion systems based on ECG and PPG, including cuffless BP estimation, stress detection, HR, and HRV monitoring. Finally, Section 6 and Section 7 summarize the important points of this comprehensive review and discuss the limitations and challenges of wearable multimodal fusion application systems based on ECG and PPG.

This review follows a structured narrative approach to identify and analyze recent developments in wearable multimodal fusion systems based on ECG and PPG for physiological monitoring applications. Relevant studies were collected from major scientific databases, including PubMed, IEEE Xplore, Scopus, and Web of Science, using keywords such as ‘wearable ECG,’ ‘wearable PPG,’ ‘ECG-PPG fusion,’ and ‘physiological monitoring.’ Approximately 300 research articles were initially screened and reviewed during the literature search process. The inclusion criteria focused on studies related to wearable or ambulatory monitoring systems, works involving ECG, PPG, or their combined use, and applications such as BP estimation, stress detection, and HR analysis. Most of the selected studies were published within the last five years. However, important and widely cited studies from the past ten years were also included to provide a broader perspective. The exclusion criteria included non-wearable hospital-based systems, purely theoretical studies without practical validation, and duplicate publications. Finally, the selected studies were organized based on signal type, device type, and application area to ensure a clear and systematic presentation.

## 2. Theoretical Background of ECG and PPG Signals

This section provides theoretical background information along with an in-depth explanation of the characteristics of ECG and PPG signals. It is hoped that this review will give the researchers the opportunity to utilize these characteristics with real-world applications in future research.

### 2.1. Electrocardiogram Signal Characteristics

**Figure 1:**
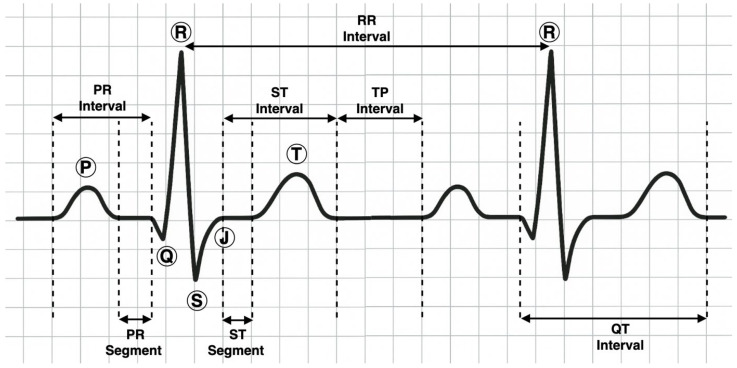
Schematic representation of a typical electrocardiogram (ECG) waveform illustrating the main components (P wave, QRS complex, and T wave) and clinically relevant time intervals and segments, including the PR interval and segment, ST segment and interval, TP interval, QT interval, and RR interval.

‘ECG’ stands for electrocardiogram, or electrocardiograph, and is also abbreviated as ‘EKG’ in some countries. An ECG can be simply defined as a waveform produced by the heart that shows the electrical activity of the heart over time, with electrodes applied to the skin, and calculates the potential difference between the electrodes [20,21,22]. Electrical impulses that cause the heart muscle to contract regulate the heart’s function, and these impulses create detectable voltage changes on the skin’s surface. These voltage variations are captured by an ECG as a waveform, which consists of elements such as the P wave (represents depolarization of atria), the QRS complex (represents depolarization and contraction of ventricles), and the T wave (represents repolarization of ventricles) [20,22]. The P wave is round in appearance and shows a small upward deflection before the QRS complex [20,22,23]. The duration of the P wave is normally less than 0.12 s (120 ms), and the amplitude is less than 2.5 mm [24,25]. In the QRS complex, the Q wave indicates a small downward deflection before the R wave, and the R wave represents a large upward deflection. The S wave shows a deflection back downward after the R wave [20,23,26]. The appearance of this QRS complex is a sharp and narrow spike. The duration of this complex is typically in the range of 0.06 to 0.10 s (60–100 ms), but the average QRS interval is less than 0.12 s. Atrial repolarization occurs simultaneously but is obscured by the QRS complex [27,28]. The appearance of the T wave is a broad, rounded, and typically upright waveform that occurs after the QRS complex [20,23]. The duration of the T wave is variable but usually ranges from 0.10 to 0.25 s [27]. A schematic illustration of a typical ECG waveform is shown in Figure 1.

Beyond the P wave, QRS complex, and T wave, which are described in depth above, several key features or characteristics of the ECG can be identified. The first of these is the PR interval, which is the time from the beginning of the P wave to the beginning of the QRS complex. It reflects conduction through the atrioventricular node (AV node). That is, it represents the time required for an electrical impulse to travel from the atria to the ventricles through the atrioventricular (AV) node and the bundle of His. The normal PR interval value is less than 0.20 s [20,27]. The PR segment is the flat, typically isoelectric horizontal segment of the ECG signal between the end of the P wave and the beginning of the QRS complex [21,29]. The junction point, or J point, of the ECG signal is the point where the QRS complex connects to the ST segment. It is considered to represent the near end of depolarization and the beginning of repolarization as determined by the ECG [29]. The ST segment of the ECG signal can be identified as the flat, isoelectric portion between the end of the S wave, i.e., the J point, and the starting point of the T wave. This ST segment can be identified as the period when the ventricles are completely activated [20,23,29]. Another characteristic of an ECG signal, the QT interval, is measured by measuring the time from the beginning of the Q wave of the QRS complex to the end of the T wave. This interval represents the time it takes for ventricular depolarization and repolarization. That is, the time from effective ventricular contraction to effective relaxation is referred to as ventricular systole [27,29]. The RR interval in an ECG signal is the time between two consecutive R waves or QRS complexes. It is essential for calculating HR [21,27].

In addition to the structural description of ECG waveforms, accurate identification and delineation of key components such as the P wave, QRS complex, and T wave play a fundamental role in physiological signal analysis [30]. Numerous studies have focused on automated ECG delineation techniques to precisely locate fiducial points, including R-peak detection, QRS onset and offset, and T-wave boundaries [30,31,32]. Classical approaches include derivative-based methods, thresholding, and the Pan–Tompkins algorithm, while more advanced techniques employ wavelet transforms, adaptive filtering, and ML models to achieve robust detection under noisy and motion-prone conditions typical of wearable systems [30,32]. These delineated features form the basis for extracting clinically relevant parameters such as RR intervals, QT intervals, and ST segment deviations, which are essential for applications including arrhythmia detection, HRV analysis, and cardiovascular risk assessment [21,27,29,32]. In the context of ECG-PPG multimodal fusion, accurate ECG delineation is particularly critical, as it directly affects the estimation of timing-based features such as pulse arrival time (PAT) and pulse transit time (PTT) [33,34]. Therefore, ECG waveform delineation is not only a preprocessing step but also a key component that directly influences the accuracy and reliability of downstream physiological monitoring and multimodal fusion systems.

### 2.2. Photoplethysmography Signal Characteristics

**Figure 2:**
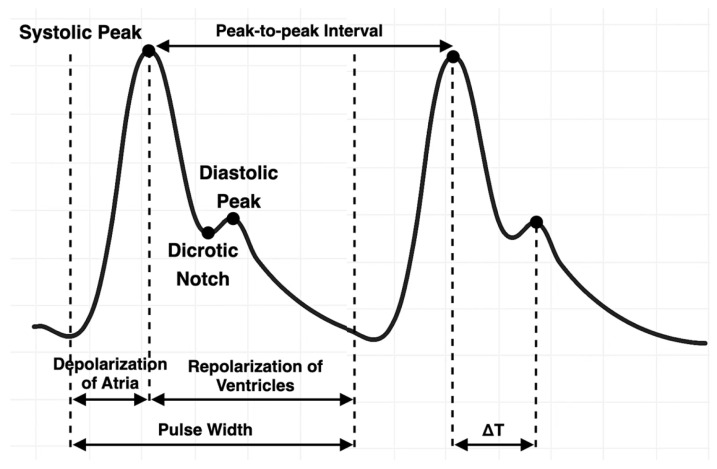
Schematic representation of a typical photoplethysmography (PPG) waveform illustrating key morphological features, including the systolic peak, dicrotic notch, and diastolic peak. The figure also highlights physiologically relevant temporal parameters such as the peak-to-peak interval, pulse width, and characteristic time differences (ΔT), along with waveform phases associated with atrial depolarization and ventricular repolarization.

PPG can be simply defined as an optical measurement technique used to detect changes in blood volume in the microvascular bed of tissues, typically caused by the cardiac cycle [35]. A light-emitting diode (LED) and a photodetector, usually with infrared, red, or green light, are commonly used to record a PPG signal. In PPG, light emitted by diodes causes changes in the amount of light that is absorbed or reflected by the pulsatile flow of blood, thereby measuring blood flow. The most recognizable waveform characteristic of the resulting wave is the peripheral pulse, which represents the heartbeat; i.e., a synchronous waveform is formed for each heartbeat [22,35,36]. As mentioned earlier, there are two PPG configurations, which are transmission mode, where light passes through tissue, and reflection mode, where light is reflected from tissue [36]. The main elements or features of a PPG waveform that show how the volume of blood in peripheral blood vessels fluctuates with each heartbeat are the systolic peak, diastolic peak, and dicrotic notch. The systolic rising edge of the anacrotic phase is caused by the expansion of the arterial system due to blood flow. The systolic peak represents the maximum blood volume in the peripheral arteries following ventricular contraction. The amplitude of the systolic peak is related to the stroke volume because the rate of expansion is related to the contraction of the heart. The appearance of this peak is the first major upward peak in the PPG waveform. It occurs shortly after the QRS complex in the ECG [21,22,36,37]. A schematic illustration of a typical PPG waveform is shown in Figure 2.

The dicrotic notch represents a brief rise in pressure after the closure of the aortic valve. The reason for the dicrotic notch is that when the left ventricle finishes ejecting blood, the aortic valve closes, and a small backward pressure wave causes a slight dip (notch) in the waveform. It occurs during early diastole, and its location and timing are influenced by arterial stiffness [36,37,38]. The diastolic peak is a secondary upward bump in the PPG waveform after the systolic peak and the dicrotic notch, and it represents a reflected pressure wave from peripheral arteries, such as at arterial bifurcations or resistance points. It occurs during early to mid diastole, which means it typically appears 0.1 to 0.3 s after the systolic peak [36,37,39]. Beyond the systolic peak, diastolic peak, and dicrotic notch, which are described in depth above, several key features or characteristics of the PPG can moreover be identified. The peak-to-peak interval of a PPG wave is the distance between two subsequent systolic peaks. Furthermore, the duration between the systolic and diastolic peaks (ΔT) correlates with the time required for the pulse wave to travel from the heart to the periphery and return. The time it takes from the beginning of PPG to its systolic peak is called the systolic phase (systole period) or the crest, which is the depolarization of the atria. The repolarization of the ventricles represents the diastolic phase. The summation of these two phases is the pulse width of the heart [21,22,37].

In addition to the morphological description of PPG waveforms, extensive research has focused on extracting meaningful features from PPG signals for physiological monitoring applications. PPG feature extraction can generally be categorized into time-domain, frequency-domain, and time–frequency-domain approaches [39,40,41,42,43]. Time-domain features include systolic peak amplitude, pulse width, rise time, and temporal intervals such as peak-to-peak intervals and systolic–diastolic time differences, which are widely used for heart rate estimation, vascular assessment, and pulse transit analysis [40]. Morphological features, including the shape of the systolic upstroke, dicrotic notch prominence, and diastolic decay characteristics, provide valuable information about arterial stiffness and cardiovascular function. In the frequency domain, spectral features derived from Fourier analysis are used to assess periodic components related to cardiac and respiratory activity [41,42]. Furthermore, time–frequency methods such as wavelet transform have been increasingly applied to capture the nonstationary characteristics of PPG signals, enabling more robust feature representation under dynamic conditions [39,41,42]. In recent studies, ML and deep learning (DL) techniques have also been employed to automatically learn discriminative features from raw or minimally processed PPG signals, reducing reliance on handcrafted features while improving performance in applications such as BP estimation, stress detection, and HRV analysis [40,41,43,44]. These feature extraction strategies play a critical role in enhancing the accuracy and robustness of both standalone PPG-based systems and ECG-PPG multimodal fusion frameworks.

Accurate monitoring techniques are essential for reliably identifying the intrinsic characteristics and features of the ECG and PPG signals described in this section. Accordingly, these aspects are considered in the next section.

## 3. Wearable Monitoring Technologies for ECG and PPG

**Figure 3:**
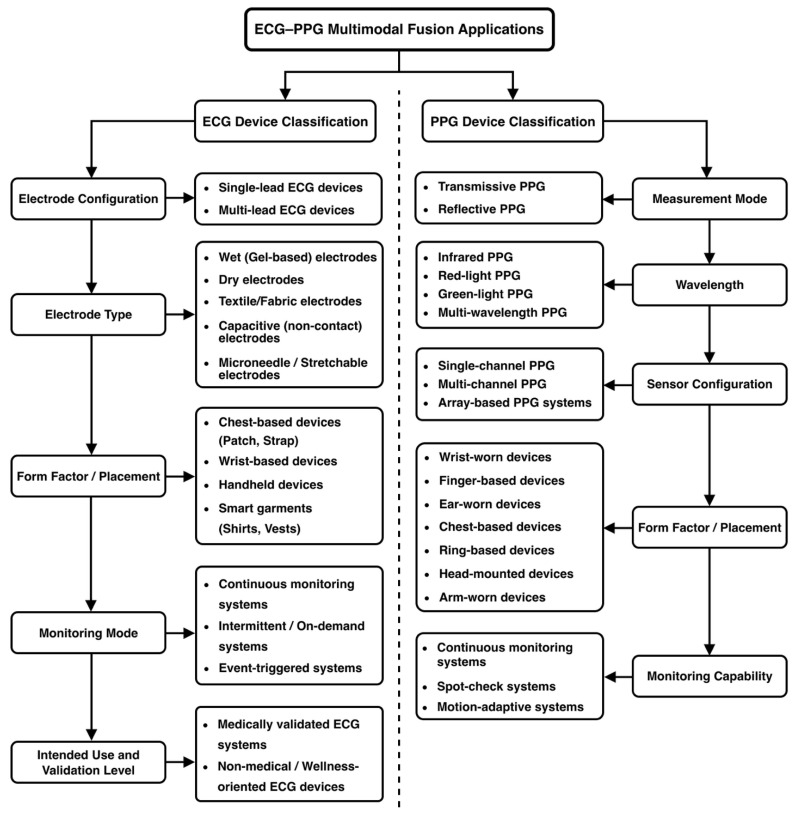
Schematic illustration of the categorization of wearable ECG and PPG monitoring devices according to device type, sensing modality, and anatomical placement.

This section reviews recent wearable monitoring techniques for ECG and PPG signal acquisition. These techniques provide the foundation for the development of next-generation wearable monitoring devices based on ECG and PPG. Physiological signal-acquisition devices that are non-invasive, portable, body-attached or body-integrated, capable of continuous or semi-continuous monitoring, and designed for real-world or daily life use are considered “wearable devices.” Clinical bedside monitors and large diagnostic systems were excluded from this review, as they do not meet the criteria for wearable devices, despite their ability to acquire physiological signals. Figure 3 presents a categorization of wearable ECG and PPG monitoring devices for multimodal fusion applications.

**Figure 4:**
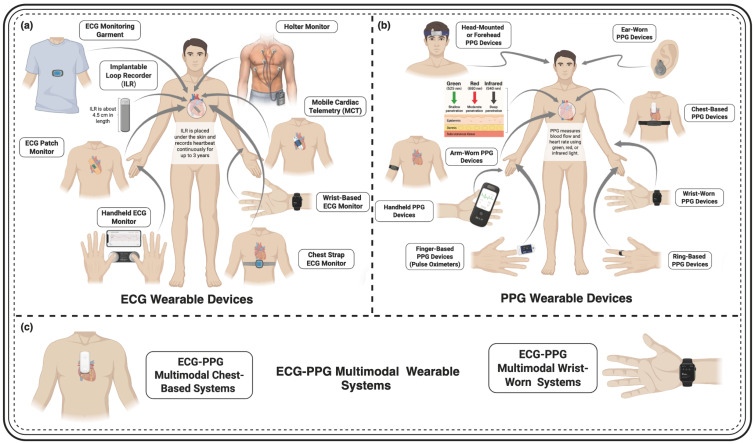
Overview of wearable ECG and PPG devices and their integration into multimodal systems. (**a**) ECG wearable devices; (**b**) PPG wearable devices; (**c**) ECG-PPG multimodal wearable systems, including chest-based and wrist-worn configurations.

ECG wearable devices discussed in this review include ECG patches, wrist-based ECG monitors, chest-strap ECG devices, smart ECG garments, Holter monitors, and handheld ECG monitors that can be seen in Figure 4a. PPG wearable devices discussed in this review include wrist-worn devices, finger-based devices, ear-worn devices, chest-based PPG devices, ring-based devices, and arm- or head-mounted devices, as shown in Figure 4b. Multimodal wrist-worn and chest-based systems (Figure 4c) facilitate the simultaneous acquisition of ECG and PPG signals using a single device for ECG-PPG multimodal fusion applications. One or a combination of these ECG and PPG wearable devices can be utilized for ECG-PPG multimodal fusion applications, depending on several key factors, including signal quality and stability, device placement and form factor, synchronization capability between modalities, power consumption and computational constraints, user comfort and wearability, and the specific requirements of the target application, such as cuffless BP estimation, stress monitoring, or HRV analysis. Most wearable devices developed in the past five years capable of acquiring physiological signals, such as ECG, PPG, both signals, or multimodal combinations including at least one of these, were considered in this review. The discussion begins with recent ECG wearable monitoring technologies.

### 3.1. ECG Wearable Monitoring Technologies

**Figure 5:**
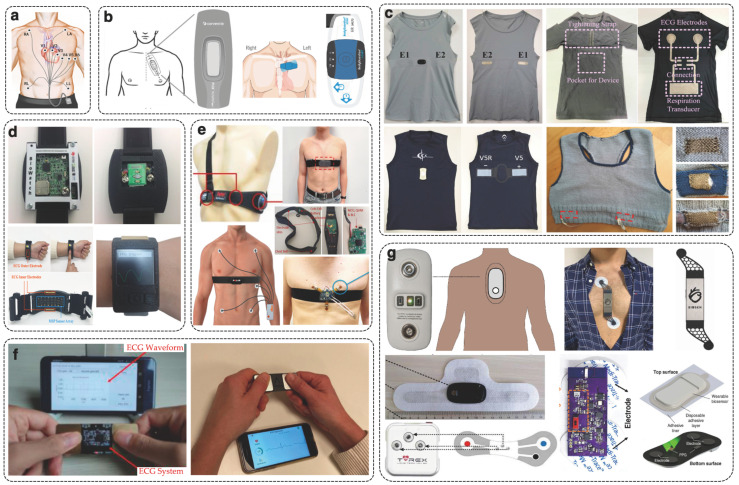
Summary of recent ECG wearable devices: (**a**) Holter monitors [50]; (**b**) Mobile cardiac telemetry (MCT) systems [53,55]; (**c**) ECG monitoring garments [30,46,47,58]; (**d**) Wrist-based ECG monitors [60,61,62]; (**e**) Chest-strap ECG monitors [64,65,66,67,68]; (**f**) Handheld ECG monitors [69,70]; and (**g**) ECG patch monitors [31,71,72,73,74,75].

In clinical electrocardiography, multi-lead systems consisting of 3 to 12 leads are typically used for ECG acquisition. In contrast, wearable applications commonly employ single-lead or dual-lead ECG configurations, with electrodes placed on the wrist or chest. ECG-based wearable devices employ a variety of electrode technologies, including wet (gel-based) electrodes, dry electrodes such as metal dry electrodes, conductive polymer electrodes, and carbon-based electrodes, textile or fabric electrodes, microneedle electrodes, and stretchable, skin-conformal electrodes [22,45,46,47]. In these systems, the ECG signal is obtained from the voltage difference measured between the electrodes [22,45]. Figure 5 summarizes recent wearable ECG devices, where most systems acquire only ECG signals, while some also incorporate PPG sensing, and others support multimodal signal acquisition using a single integrated device.

ECG wearable devices, including Holter monitors, mobile cardiac telemetry (MCT) systems, ECG monitoring garments, wrist-based ECG monitors, chest-strap ECG monitors, handheld ECG monitors, and ECG patch monitors, can be used for ECG acquisition. The Holter monitor is a portable, continuous ECG recording device that uses three to twelve leads to record heart activity over a 24- to 72 h period, covering both daytime and nighttime periods, including rest and physical activity [48,49]. Electrodes of the Holter monitor are affixed to the chest and linked to a portable recorder, which is fastened to a belt or strap in this apparatus. It continually captures ECG signals for professionals to analyze offline. The primary advantages of the Holter monitor are its ability to continuously monitor ECG for 1–3 days and high diagnostic accuracy. Additionally, the ECG can be recorded during real-world activities. Bulky and intrusive, unable to transmit data in real-time, and with limited recording duration due to battery and memory constraints are the main limitations of this device [48,50,51,52]. Figure 5a shows the Holter recording apparatus. Peng Zhang’s study utilized ECG signals acquired from 24 h Holter monitoring for the application of automatic AF detection.

Mobile cardiac telemetry (MCT) is a wireless technology-based real-time ECG monitoring system that sends data to a remote monitoring centre, which can be seen in Figure 5b. Corventis, which is a pioneer in the field of mobile patient monitoring, has developed a wearable device called NUVANT. It’s a mobile cardiac telemetry system that provides near real-time feedback on cardiac arrhythmias and other vital signs, including HR [53,54]. A similar MCT has been designed, called the BodyGuardian Heart (BGH), and has been used in several studies to monitor ECG [55,56]. The key advantages of this device are real-time analysis and alerts, and longer wear time, up to 30 days. The primary drawback of this device is that it is very expensive compared to other methods. Furthermore, it needs wireless connectivity and may require manual recharging [53,55,57]. The studies by Jonathan M. Engel et al. [53] and Mark E. Willcox et al. [55] used only ECG signals acquired from a wearable mobile cardiac telemetry system for continuous monitoring and detection of cardiac arrhythmias.

ECG smart clothing, or ECG monitoring garments, including shirts, vests, or tank tops, has flexible or textile-based dry electrodes placed on them that continually record biosignals, including ECG readings. Conductive fabrics are commonly used for the electrodes in these devices, which are printed or sewn onto clothing [30]. Following skin contact, the fabric-based dry electrodes gather ECG data similarly to that of conventional gel electrodes. This method is very comfortable to wear and reusable. Therefore, it is suitable for continuous, long-duration, and daily life monitoring applications [30,58,59]. These devices have some limitations, such as the output signal may change with movement and sweating, and electrode-skin contact may not be accurate. In addition, they may require a tight fit for optimal performance and may require washing precautions [30,47]. Several ECG monitoring wearables that have been designed and developed through various studies are shown in Figure 5c. A stretchable single-lead ECG garment was used by Yuxiang Bu et al. [46]. ECG signals were acquired via flexible silver-coated dry electrodes embedded in a smart garment for long-term cardiac monitoring with reduced motion artifacts. The proposed system of Kristel Fobelets et al. [47] utilized ECG signals acquired via knitted textile electrodes integrated into relaxed-fitting garments for wearable cardiac monitoring under static and mild ambulatory conditions. The Shuenn-Yuh Lee et al. [30] system used ECG and respiratory signals acquired via smart clothing for long-term health monitoring and real-time analysis of cardiac activity and breathing patterns. Tsukada et al. [58] developed a system that employs single-lead ECG signals acquired via textile-based wearable electrodes integrated into garments for continuous cardiac monitoring during daily and ambulatory conditions.

The user contacts the sensor or crown with a finger while the wrist-based ECG monitor is turned on. Then it forms a closed circuit across the arms. These devices are worn like a typical watch, so users may always access cardiac monitoring features without carrying or attaching extra equipment [60,61]. It is noteworthy that these wearable devices can detect signs of common cardiac disorders, such as atrial fibrillation (AF), even before symptoms worsen. Additionally, providing early warnings like this helps users seek medical attention sooner, which can help them avoid more serious problems [62]. The limitations of these wrist-based ECG monitors include a single-lead ECG and only manual activation. Furthermore, the results may lack precision for subtle abnormalities due to the motion artifacts [61,62,63]. Figure 5d shows the wrist-based ECG monitors. The proposed systems of Hanvit Kim et al. [61] and Thomas et al. [60] utilized both ECG and PPG signals acquired via a wrist-worn wearable device for pulse transit time–based BP estimation and continuous physiological monitoring. Yongbin Lee et al.’s [62] system employed PPG and ECG signals acquired from a wrist-worn wearable platform for continuous HR monitoring and cooperative detection of AF.

The chest strap ECG monitor is a belt-like wearable worn around the chest, commonly used for health (fitness) and sports monitoring [64]. This device has two electrodes built into the strap that pick up heart signals and transmit the ECG to a paired device, such as a computer, in real-time via Bluetooth or any other technology [65]. The benefits of this device include its compactness, lightness, washability, and accuracy in measuring HR during physical activity [64,65,66].

Additionally, it can wirelessly connect with other devices [64,65]. Some chest strap ECG monitors provide HR, not a full ECG, and this device can be used for short-term use only [64,66,67]. The recently used chest strap ECG monitors can be seen in Figure 5e. The system of Anny Maza et al. [64] utilized ECG-derived RR interval signals acquired via a low-cost wearable chest-strap device for reliable estimation of short-term and ultra-short-term HRV in emotion-related physiological monitoring. Denice Gerardo et al.’s [65] proposed system utilized single-lead ECG signals acquired via a wearable chest-based device for accurate R-peak detection and real-time HR monitoring. The proposed system of Xiaoman Zhang et al. [66] employed only ECG signals acquired through wearable chest-strap textile electrodes for continuous physiological monitoring and exercise intensity assessment. The system of Daljeet Kaur Saggu et al. [67] utilized single-lead ECG signals acquired via a chest strap with dry electrodes for long-term ambulatory monitoring and detection of cardiac arrhythmias. Dheman et al. [68] developed a system that utilizes ECG signals acquired via a wearable chest-strap sensor for continuous cardiac activity monitoring and long-term ambulatory health assessment.

The handheld ECG monitor is a small, single-lead electrocardiogram portable device that allows both doctors and patients to record a brief ECG, typically 30–60 s, by touching metal electrodes with fingers for monitoring cardiac arrhythmias [70]. It captures an ECG tracing similar to that of a Lead I, and the data is either retained or sent to mobile apps via Bluetooth. This device is convenient and easy to use, requiring no skin preparation or conductive gels [69,70]. Furthermore, it’s affordable and broadly accessible for wearable personalized healthcare applications. It delivers an immediate, high-quality ECG preview and analysis. The main limitations are that it only supports manual activation and offers [69,70]. Several recently developed handheld wireless ECG recording systems can be seen in Figure 5f. Jianchan Liao et al. [69] employed ECG signals acquired from a handheld wireless wearable device using printed metal-plate electrodes for continuous cardiac monitoring and personalized healthcare applications. The system of M. Patrick Witvliet et al. [70] utilized single-lead ECG signals acquired using a handheld wearable device for the detection and monitoring of cardiac rhythm and conduction disorders, particularly AF.

Currently, the most popular wearable ECG monitoring is the ECG patch. An ECG patch is a wearable adhesive patch that continuously records an ECG from one or two leads, typically recording for one to fourteen days. These devices stick to the chest with no wires, have an internal battery, and have memory for continuous recording [74]. Furthermore, some models are connected to local storage or a smartphone via Bluetooth for real-time streaming [72]. The key advantages of this patch are that it is compact, wireless, and comfortable [72,75,76]. Additionally, it provides good patient compliance and continuous long-term monitoring [72,77]. Recently developed wearable ECG monitoring patches can be seen in Figure 5g. John A. Berkebile et al.’s [71] proposed system utilized multimodal physiological signals, including ECG, PPG, and SCG, acquired from a wearable chest patch for remote monitoring and detection of cardiovascular autonomic dysfunction in synucleinopathies. In Bardia Baraeinejad’s [72] study, single-lead ECG signals collected via an ultralow-power wearable patch were employed for continuous cardiovascular monitoring and AI-assisted arrhythmia detection in remote healthcare systems. The study of Dakun Lai et al. [31] employed single-lead ECG signals acquired from a wearable dry-electrode patch to enable continuous monitoring and automated detection of AF in real-life scenarios. In the study by Minsoo Yeo et al. [73], a multimodal wearable patch acquired ECG and PPG signals to facilitate continuous monitoring and data-driven prediction of physiological stress using PAT and HRV features. In the study by Weilun Li et al. [74], a wearable instrumentation patch was employed to acquire biopotential signals, including ECG and EEG, for continuous, motion-robust monitoring and real-time health assessment in ambulatory and remote healthcare applications. The system of Glenn Fernandes et al. [75]. employed ECG and respiratory-related signals acquired from a patch-type wearable device to enable automated detection of sleep-related respiratory events and accurate estimation of the apnea–hypopnea index (AHI).

Beyond external wearable ECG devices, implantable loop recorders (ILR) offer an alternative approach for long-term continuous ECG monitoring. An ILR is a subcutaneously implanted device that provides long-term, up to 3 years, continuous ECG monitoring for various cardiovascular conditions. It is implanted under the skin near the chest to continuously record and store ECG episodes and send alerts wirelessly to cardiologists or physicians for a variety of clinical syndromes. The main advantages of this method are an extremely long monitoring duration, automatic event detection, and remote physician access. But this method requires a minor surgical procedure, is high-cost, and cannot be removed easily [78,79].

### 3.2. PPG Wearable Monitoring Technologies

**Figure 6:**
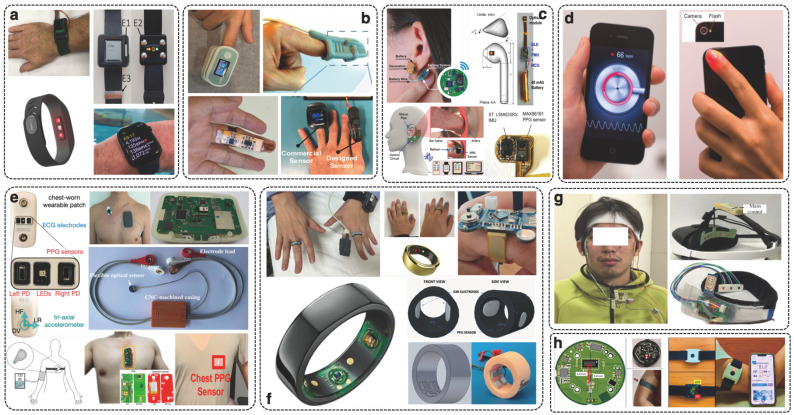
Summary of recent PPG wearable devices: (**a**) Wrist-worn PPG devices [80,87,88,90]; (**b**) Finger-based PPG devices (pulse oximeters) [81,91,94,95]; (**c**) Ear-worn PPG devices [43,82,96,97]; (**d**) Smartphone camera-based handheld PPG monitoring [98]; (**e**) Chest-Based PPG devices [102,103,105,106,107,113]; (**f**) Ring-Based PPG devices [83,84,108,109,110,111]; (**g**) Head-mounted or forehead PPG devices [112,114,115]; and (**h**) Arm-worn PPG devices [116,117].

PPG is a non-invasive optical method that measures changes in blood volume in the microvascular bed of tissue (hemodynamic information) using a light source and a photodetector [22]. PPG-based wearable devices employ a variety of sensor technologies, including transmissive PPG and reflective PPG sensors that operate using different light wavelengths, such as green, red, and infrared, as well as multi-wavelength configurations [80,81,82,83,84]. Frequently integrated in modern PPG-based devices, including watches, rings, and fitness bands that have continuous recording capabilities, even when the wearer is moving their wrist, finger, or forehead, which provides a more reusable and comfortable design [45,85,86]. Figure 6 summarizes recent wearable PPG-based devices, where the majority are designed for PPG signal acquisition, while a subset of devices also support multimodal sensing by capturing additional physiological signals beyond PPG.

PPG wearable devices, including wrist-worn PPG devices, finger-based PPG devices (pulse oximeters), ear-worn PPG devices, smartphone camera–based handheld PPG devices, chest-based PPG devices, ring-based PPG devices, head-mounted or forehead PPG devices, and arm-worn PPG devices, are typically used for PPG signal acquisition. Wrist-worn devices such as wristwatches and wristbands are commonly recognized as wearable devices. These devices emit infrared or green light onto the skin through a light transmitter, and the reflected light is measured by a photodiode, and the changes in the reflection correspond to the PPG pulse waves [80]. The main advantages of these devices are ease of wear, convenience of long-term monitoring, always-on operation, and increased accuracy with motion correction. The main limitations of these devices are the occurrence of motion artifacts during physical activity and low accuracy on cold skin or dark skin tones, and they may also be unreliable for clinical diagnosis [87,88,89]. Recent examples of wrist-worn devices, including watches and wristbands, can be seen in Figure 6a. The system of Justinas Bacevicius et al. [87] employed combined PPG and six-lead ECG signals acquired from a wrist-worn device for continuous AF detection and reliable cardiac rhythm verification in mobile health applications. The study by Ben O’Grady et al. [88] utilized PPG signals along with tri-axis accelerometer data for real-time HR estimation and motion artifact suppression in wearable health monitoring applications. Selder et al.’s [90] study employed wristband-derived PPG signals for long-term cardiac rhythm monitoring and automated detection of AF using a standalone algorithm.

When discussing finger-based devices, the pulse oximeter is a key device that can be included in this discussion. This device, which is widely used in modern medicine, is equipped with a transmissive PPG, in which light passes through the finger. That is, two light-emitting diodes of different wavelengths transmit light through the arterial blood of a finger, and the transmitted light is detected with a photodiode. This device can continuously monitor the active oxygen saturation of hemoglobin in arterial blood and assess HR [81,91,92,93]. The main advantages of this device are its high accuracy and wide use in clinical and home settings. In addition, it has minimal signal noise in stationary environments and a low cost of hemodynamic monitoring. The drawback of this device is that it is not wearable for long durations, unlike a smart ring. Furthermore, it’s sensitive to motion and finger positioning and is limited in multi-parameter monitoring [91,92,93]. Several recently designed pulse oximeters and finger-based devices can be seen in Figure 6b. Timm et al. [81] proposed a non-invasive optical sensing system that utilized PPG signals for continuous real-time monitoring of hemodynamic and cardiovascular parameters in clinical and healthcare applications. PPG signals acquired from a low-cost wearable pulse oximeter were utilized by Sandra Viciano-Tudela et al. [91] for continuous remote monitoring of vital physiological parameters, including SpO_2_, pulse rate, and respiratory rate, in e-health applications. In the study by Sadaghiani et al. [94], PPG signals acquired under ambient light are utilized in a wireless wearable finger patch for continuous monitoring of vital signs, including HR and BP. Mazandarani et al. [95] proposed an ultralow-power wearable sensing system that employs PPG signals for continuous monitoring of vital physiological parameters such as HR and blood oxygen saturation.

Research has shown that the earlobe has a much less sensitive PPG signal to perfusion changes compared to the limbs. Therefore, ear-worn devices have become very popular today, and these devices detect reflective PPG in the ear canal or earlobe [96]. Furthermore, these ear-worn devices are more comfortable for long-term wear, and they detect better waveforms close to central arteries [82,96]. However, these devices are typically used in specific contexts, which is already difficult to see in clinical practice. The reason this device is not widely available is that it is still largely in the early stages of development. But among the ear-worn devices, research information can also be seen on fashion designs such as PPG earrings, which are designed to target the general public [43]. The accuracy of the output varies across models, which is another limitation [97]. Several of the ear-work devices described above are summarized in Figure 6c. Montanari et al. [82] developed EarSet, a multimodal dataset acquired using earbud-integrated 3-channel PPG sensors and 6-axis IMUs, enabling systematic analysis of motion artifacts on in-ear PPG signals for robust estimation of cardiovascular parameters such as HR and HRV in wearable devices. Boukhayma et al. [96] developed an earbud-embedded micro-power optical sensor that utilizes PPG signals acquired from the ear to enable accurate and continuous heartbeat monitoring for wearable health applications. Xue et al. [43] proposed a wireless, ear-worn wearable system that employed reflective PPG signals from the earlobe to enable accurate and continuous HR monitoring under daily life and exercise conditions. In the study by Bui et al. [97], PPG signals acquired via an ear-worn wearable device were utilized for continuous and unobtrusive BP monitoring in ambulatory and daily life healthcare applications.

Handheld PPG devices are small, portable devices operated by holding them in the hand or placing a finger on a sensor. Transmissive or reflective PPG is used in this method. The main advantages are that they are highly portable, affordable, and easy for home or field use. However, they don’t have continuous monitoring. These devices’ poor perfusion and sensitivity to movement are other drawbacks [98,99]. A smartphone camera-based handheld PPG monitoring system can be seen in Figure 6d. Chan et al. [98] proposed a smartphone-based PPG sensing application that enabled non-invasive acquisition of pulse waveforms for reliable AF detection and large-scale cardiovascular screening in primary care environments.

Chest-based systems with adhesive patches or wearable chest straps are commonly used for ECG monitoring, but chest-based PPG monitoring is less commonly used. However, few studies have reported data that have integrated reflective PPG sensors into adhesive patches or wearable chest straps in chest-based systems. Reflective PPG employs a photodetector and light on the same side that faces the skin, so the devices are comfortable for the user for short-term monitoring [100]. These sensors track variations in the microvasculature of the chest as pulsatile blood flow absorbs and reflects light, and these devices maintain stable contact with human skin while monitoring [100,101]. But this device is less comfortable for long-term casual use since it requires a tight attachment to the chest, such as a strap or adhesive patch. So, these chest-based devices are not consumer-friendly but can be effective for clinical settings [102,103,104]. Chest-based devices, including adhesive patches and wearable chest straps, that have been developed in recent studies can be seen in Figure 6e. Li et al. [102] developed an IoT-enabled wearable system that simultaneously acquired multimodal physiological signals, including ExG (ECG/EEG/EMG), PPG, and bioimpedance (BioZ), for continuous full-body health monitoring and remote chronic disease management. Marzorati et al. [103] proposed a multimodal chest-based wearable device that combines PPG and PCG signals for continuous and non-invasive BP monitoring using PAT-based modeling and cardiovascular signal processing. Chan et al. [105] proposed a multimodal chest-mounted wearable system that extracts respiration-related information from ECG, PPG, and SCG signals and employs modality-attentive fusion with U-Net-based denoising to achieve accurate respiratory rate estimation under motion-intensive conditions such as walking. Bellier et al. [106] developed a chest-worn wireless wearable system that integrates multimodal signals, including ECG, PPG, bioimpedance, temperature, and accelerometry, to enable continuous monitoring of vital signs and real-time Early Warning Score (EWS) calculation in hospitalized patients. Park et al. [107] developed a chest-based wearable system integrating PPG and phonocardiography (PCG) signals to enable multi-mode health monitoring, including HR measurement, digital stethoscope functionality, and vascular transit time (VTT)-based BP estimation.

Researchers have focused on obtaining PPG using ring-based devices. Similar to other methods, ring-based devices use PPG sensors, which are positioned on the inner surface of the ring where the user’s finger touches the skin, increasing the comfort of the person wearing the ring to take measurements. LEDs emit light into finger tissues when worn, and the photodetector measures the reflected light. This approach eliminates the need for additional body-worn devices and captures all signals through the ring [108]. The positive aspects of this ring-based device are its high wearability, convenience, and continuous monitoring, which includes being able to receive a signal even while sleeping without user action [109]. In addition, it provides good signal stability, and output signals can be easily captured into a smartphone or other electronic device [109,110]. Due to their compact size, the battery life of these devices is limited in continuous tracking. Furthermore, they limit the use of high-power sensors, and these are the limitations of this device. However, several studies have been conducted to capture various types of biosignals, including PPGs, in recent years [83,108,110,111]. Figure 6f shows the developed smart rings for PPG detection through those studies. Guo et al. [83] proposed a smart ring-based multimodal sensing framework that integrates PPG and SpO_2_ signals with DL to achieve accurate, continuous detection of obstructive sleep apnea-hypopnea syndrome (OSAHS) in sleep monitoring applications. Gabriele Volpes et al. [84] proposed a wearable ring-shaped device that acquired ECG, PPG, and galvanic skin response (GSR) signals to enable continuous physiological monitoring and assessment of cardiovascular activity and stress conditions. Valenti et al. [108] developed a wearable ring-shaped multisensor system that acquires PPG and GSR signals to enable real-time assessment of physiological stress and blood oxygen saturation (SpO_2_) for continuous health monitoring. Kheirinejad et al. [109] employed a wearable ring integrating PPG, inertial sensing, and temperature measurements to support continuous sleep monitoring and quantitative analysis of smartphone usage effects on sleep quality and physiological recovery. Liu et al. [110] proposed a PPG-based smart ring that acquires multi-wavelength reflective PPG signals to support continuous, non-invasive monitoring of vital signs and advanced health analytics, including activity tracking, stress evaluation, and sleep staging. Osman et al. [111] developed a single-channel bioimpedance (Bio-Z) ring sensor that captures arterial blood flow signals to facilitate continuous and non-invasive BP estimation using ML models.

The reflective PPG used in other parts of the body, such as the wrist, is used in the head-mounted or forehead devices. Where the photodetector and light emitter are positioned on the same side, facing the skin, and these devices are best used for long-term measurement of signals as well as in dynamic situations [112]. The forehead or head is a good place to use light to detect changes in blood volume because of its extensive network of capillaries and tiny blood vessels.

However, it is important to properly position the used electrodes [112,115]. These head-mounted or forehead accessories can be used in multimodal biosensing and be developed for various practical applications, such as virtual reality gaming and emotion recognition [114,115]. Although these devices are easy to wear, they are not comfortable for long-term use, which can be identified as a key limitation [115]. Figure 6g shows some examples of head-mounted or forehead devices. Liu et al. [112] employed reflective PPG signals from a wearable forehead pulse oximeter and leveraged supervised classification methods, including SVM and CNN, to evaluate signal quality and enhance the accuracy of SpO_2_ monitoring in motion-prone environments. Otsuka et al. [114] utilized PPG signals acquired via a forehead-mounted sensor embedded in a virtual reality (VR) headset for continuous HR monitoring and physiological assessment during immersive gaming environments. Wan et al. [115] developed a wearable multimodal bio-signal acquisition system that simultaneously utilizes EEG, PPG, electrodermal activity (EDA), and skin temperature (SKT) signals for emotion recognition in immersive VR environments.

Researchers have successfully measured PPG with upper-arm devices during moderate-to-high levels of physical activity. The reflective PPG sensors on these wearing bands are placed on the upper arm to acquire reflective PPG signals [116,117]. Their positioning differs from that of wrist devices to minimize motion artifacts and frequently attain greater signal stability [117]. These devices are more stable in the arterial bed of the upper arm and have less motion noise than the wrist, and they are more comfortable compared with finger-based devices such as pulse oximeters [117]. Additionally, they are often more accurate than other PPG devices in motion, as demonstrated by some studies [116,117,118]. However, they may be less convenient than a smartwatch and bulky or less stylish. Some examples of arm-worn PPG devices can be seen in Figure 6h. Kinnunen et al. [116] developed a wearable upper-arm multiwavelength PPG-based sensor for continuous monitoring of HR and blood oxygen saturation in wellness, sports, and health applications. Wang et al. [117] developed a wearable cuffless BP monitoring system based on upper-arm PPG signals, utilizing a machine-learning model with one-time calibration to enable accurate long-term continuous BP estimation.

It is also important to distinguish wearable monitoring systems from clinical gold-standard measurements. Clinical ECG systems typically use multi-lead configurations such as 12-lead ECG, providing high diagnostic accuracy and comprehensive cardiac information, whereas wearable ECG devices generally rely on single- or dual-lead configurations, resulting in reduced spatial resolution [30,50,77]. For PPG-only wearable systems to achieve clinically meaningful accuracy, several conditions must be met. These include stable sensor-skin contact, effective suppression of motion artifacts, sufficient signal quality achieved through proper sensor placement, such as on the finger or ear, and the use of advanced signal processing or ML techniques [80,91,96]. Additionally, calibration and validation against reference measurements are often required to ensure reliable physiological interpretation, particularly for applications such as BP estimation [81,82,88]. As a result, while wearable systems enable continuous and real-time monitoring, they often involve a trade-off between convenience and measurement accuracy.

Although ECG and PPG wearable devices provide continuous physiological data collection, the successful integration of these inputs necessitates meticulous synchronization and preprocessing. Therefore, the next section concentrates on signal synchronization and preprocessing methodologies for ECG-PPG wearable multimodal fusion systems.

## 4. Signal Synchronization and Preprocessing for ECG-PPG Wearable Multimodal Fusion Systems

This section reviews signal synchronization and preprocessing techniques for wearable ECG-PPG multimodal fusion systems, including time alignment, motion artifact mitigation, noise suppression, analysis window size selection, and strategies for handling missing or corrupted data, all of which play a critical role in fusion performance.

### 4.1. Signal Synchronization and Time Alignment

Since ECG and PPG capture different physiological events with inherent temporal delays, accurate time alignment between the two signals is essential [119,120]. Specifically, ECG reflects the electrical activity of the heart, whereas PPG measures changes in peripheral blood volume that occur after a physiological propagation delay [120]. Applications such as cuffless BP measurement and arrhythmia detection depend on precise time alignment [62,120]. One widely used approach for time alignment is pulse transit time (PTT)–based alignment [119,121]. To achieve signal synchronization, this approach estimates and compensates for the temporal delay between the ECG R-peak and a characteristic point on the PPG waveform, such as the systolic peak or the waveform foot [119,120,122]. Timestamp-based synchronization is utilized when ECG and PPG are acquired from the same wearable device over a shared clock [71]. Cross-correlation techniques can also be employed to determine the relative delay between ECG and PPG signals by optimizing the alignment between ECG-derived heartbeats and corresponding PPG pulses [62,120,123]. Interpolation and resampling techniques can be used for time alignment when ECG and PPG signals are acquired at different sampling frequencies [73,124]. These methods adjust one or both signals to a common time base, enabling accurate synchronization.

In addition to conventional synchronization approaches such as PTT-based alignment and cross-correlation, several advanced techniques have been explored in the literature to improve synchronization accuracy under real-world conditions. Dynamic time warping (DTW) has been employed to achieve non-linear alignment between ECG and PPG signals by compensating for temporal variations caused by HRV and physiological delays [124]. Phase-based synchronization methods, including phase locking and Hilbert transform-based phase analysis, have also been utilized to capture the temporal relationship between signals at the cardiac cycle level [125,126]. Furthermore, in multi-sensor wearable systems, clock drift correction and synchronization mechanisms are essential to mitigate timing inconsistencies between independently operating sensing units, particularly in ECG-PPG-based systems, where accurate temporal alignment is required for PTT and PAT estimation [121,124]. More recently, ML-based synchronization approaches have been introduced to automatically learn optimal alignment patterns from multimodal data, offering improved robustness in complex and dynamic environments [122,124].

### 4.2. Motion Artifact Mitigation

One of the major challenges with wearable ECG-PPG devices is motion artifacts. These artifacts are caused by changes in contact pressure, muscle activity, skin deformation, and sensor displacement [31,62,72]. Artifact removal strategies include filtering-based approaches that attenuate motion-related frequency components, such as bandpass and adaptive filtering techniques [31,72,127]. In addition, signal quality indices (SQIs) are often used to assess signal reliability and exclude contaminated segments prior to fusion [120]. In accelerometer-assisted artifact reduction, inertial measurement unit (IMU) data serve as reference signals for adaptive filtering to reduce motion-related artifacts [128]. ML–based approaches are also employed for motion artifact removal, utilizing models such as convolutional neural networks or autoencoders to identify and suppress segments of the signal contaminated by motion [129,130].

In addition to these approaches, advanced signal decomposition and blind source separation (BSS) techniques have been explored to further enhance artifact mitigation performance [131,132,133]. Methods such as independent component analysis (ICA) [131,132,133,134] and principal component analysis (PCA) [131,135,136] enable the separation of motion-related interference from physiological signals by leveraging statistical independence and variance characteristics. These techniques are particularly effective in multi-channel or multimodal recordings, where motion artifacts can be isolated without significantly distorting the underlying cardiac signals [135,136]. More generally, BSS frameworks provide a systematic approach to decomposing mixed signals into independent components, enabling robust artifact suppression in dynamic and noisy environments [131,132,133,137]. These advanced methods complement traditional filtering and ML-based approaches by improving signal quality prior to feature extraction and multimodal fusion.

### 4.3. Noise Reduction Methods

A range of noise sources can degrade wearable ECG and PPG signals, including baseline wander, power-line interference, ambient light variations, and physiological artifacts related to respiration [124,125]. Noise reduction is often achieved using bandpass filtering, which is designed to preserve the physiological frequency components of ECG and PPG signals while suppressing unwanted noise. Wavelet-based denoising and baseline wander removal techniques can also be employed for effective noise reduction [138,139]. Wavelet-based denoising reduces noise while preserving signal morphology, whereas baseline wander is typically removed using high-pass filtering or polynomial detrending [127]. In addition, DL -based denoising approaches and empirical mode decomposition (EMD) techniques have been used for noise reduction in ECG and PPG signals. DL -based denoising neural networks learn noise characteristics directly from data, while EMD and its variants enable adaptive noise suppression [30,140].

Beyond their role in denoising, wavelet-based methods have been extensively utilized for feature extraction because they can represent non-stationary biomedical signals in the joint time–frequency domain [141]. Unlike conventional filtering techniques, the wavelet transform decomposes ECG and PPG signals into multiple resolution levels, enabling the isolation of clinically relevant frequency components associated with physiological events [142]. This multiresolution property allows for the extraction of discriminative features such as wavelet coefficients, energy distributions, and statistical descriptors, which have been shown to enhance pattern recognition and classification performance in physiological monitoring systems [141,143]. For example, wavelet-based decomposition has been effectively used to highlight important signal components such as QRS complexes in ECG and systolic peaks in PPG, thereby improving the detection of fiducial points and facilitating accurate physiological parameter estimation [144]. Furthermore, wavelet-based approaches have been successfully applied to identify key morphological features in PPG signals, including systolic peaks, dicrotic notches, and diastolic components, which are essential for deriving cardiovascular indices and supporting applications such as blood pressure estimation [145]. Advanced variants, such as the maximal overlap discrete wavelet transform (MODWT), further enhance signal representation and enable the extraction of robust and informative features for ML models [146]. More recently, continuous wavelet transform (CWT) has been integrated with DL frameworks to generate time–frequency representations that can be directly used for automated feature learning, reducing the need for manual feature engineering while improving classification accuracy [147]. These capabilities demonstrate that wavelet-based methods serve not only as effective noise reduction tools but also as powerful feature extraction techniques that significantly enhance the performance of ECG-PPG-based wearable monitoring and multimodal fusion systems.

While the aforementioned methods provide effective baseline noise reduction, several alternative and advanced techniques have been investigated to further enhance signal quality in wearable ECG-PPG systems. Polynomial-based smoothing methods, such as Savitzky–Golay filtering, have been applied to reduce high-frequency noise while preserving important waveform characteristics [148,149]. Nonlinear filtering techniques, including median filtering and morphological filtering, are also effective in suppressing impulsive noise and motion-induced distortions without significantly altering the underlying physiological signals [150,151]. Furthermore, beyond empirical mode decomposition, advanced decomposition frameworks such as variational mode decomposition (VMD), singular spectrum analysis (SSA), and other multi-resolution methods have been explored to enhance noise separation in non-stationary environments [150,151]. These techniques provide improved adaptability in handling complex noise patterns and complement conventional filtering and DL -based approaches, thereby enabling more robust signal preprocessing for multimodal fusion applications [150,152]. However, the selection of an appropriate noise reduction method depends on application requirements, signal quality, and computational constraints in wearable systems [150,151,152].

### 4.4. Handling Missing and Corrupted Data

Missing or damaged data segments are unavoidable in real-world wearable systems due to motion artifacts, wireless transmission issues, and sensor dropout [128,153]. Robust ECG and PPG fusion systems must be designed to handle such data imperfections, and these issues should be carefully considered by researchers during system development [138]. Data imputation techniques and modality-aware fusion strategies can be employed to handle missing or corrupted data [130,138]. Data imputation methods, such as interpolation or model-based reconstruction, are used to recover missing signal segments, while modality-aware fusion dynamically adapts the fusion process when one modality becomes unreliable [71,73,76]. Redundant feature extraction is another approach that can be used for this purpose, allowing the system to rely on alternative features when primary features are unavailable [121,154]. Segment rejection and confidence weighting can also be applied, whereby low-quality data segments are either excluded or assigned lower importance during the fusion process [129,130,138].

### 4.5. Analysis Window Size Selection

The amount of physiological information obtained from ECG and PPG signals depends on the size of the analysis window [62]. It has a direct impact on system latency, temporal resolution, computational burden, and estimation accuracy in ECG-PPG multimodal fusion systems [30,62,71]. The analysis window size in wearable applications must strike a balance between robustness to noise and motion artifacts and real-time responsiveness [138]. Based on the literature, analysis windows can be characterized by their duration and application requirements. For ECG signals, short windows in the range of approximately 2–10 s are commonly used due to their high signal-to-noise ratio and reliable R-peak detection, enabling low-latency applications such as real-time HR estimation and arrhythmia screening, including AF detection [62,72,124,155]. In contrast, PPG signals are more susceptible to motion artifacts and peripheral perfusion variability, and therefore typically require longer windows, usually ranging from 10 to 30 s or more, to stabilize pulse morphology and reduce false detections [120,124,127,138]. To improve robustness, multimodal fusion systems frequently employ window-level synchronization rather than beat-level fusion [124,127]. Fusion windows are typically constrained by the longer-duration modality, such as PPG [30,124,154]. To preserve temporal alignment for PAT and PTT computation, as well as morphological correlation analysis, ECG features are often aggregated over the same analysis window [120,122,125]. Furthermore, for higher-level applications such as stress monitoring and HRV analysis, longer windows ranging from approximately 30 s to several minutes (e.g., 1–5 min) are typically used to capture stable autonomic nervous system dynamics and improve statistical reliability [17,130,156,157]. As the analysis window size increases, memory requirements, computational cost, and power consumption also rise, necessitating careful consideration in the design of real-world multimodal fusion systems [30,62,72,127].

To provide a clearer and more structured overview of the methodologies discussed in this section, Table 1 summarizes the commonly used synchronization and preprocessing techniques in ECG-PPG multimodal fusion systems, along with their functional roles and application domains, particularly in cuffless BP estimation, stress detection, HR, and HRV monitoring. As highlighted in the table, conventional approaches such as filtering-based methods and PTT-based synchronization remain dominant across most applications, especially in cuffless BP estimation and HR monitoring. In contrast, more advanced techniques, including ML-based methods, blind source separation, and adaptive fusion strategies, are increasingly being explored to address challenges in stress detection and real-world wearable environments, where signal variability and noise are more pronounced.

## 5. Physiological Monitoring Applications of ECG-PPG Multimodal Fusion

This section will focus on specific key applications of the multimodal fusion systems based on ECG and PPG. Multimodal fusion and the ECG and PPG uses of BP estimation (cuffless), stress detection, HR, and HRV monitoring are described at length here. It is hoped that this will provide researchers with the knowledge needed to identify weaknesses and limitations in the ECG and PPG multimodal fusion systems described here and improve them or create new multimodal fusion systems.

### 5.1. Overview of ECG-PPG Multimodal Fusion

Multimodal fusion refers to the integration of data obtained from two or more modalities or systems to achieve improved performance [158,159]. Currently, obtaining results using multimodal fusion has become very popular in several fields because it has enabled a more useful, accurate, and comprehensive understanding of physiological conditions [17,62,75,119,122,123,124,125,126,140,153,154,156,157,158,160,161,162,163,164,165,166]. To highlight the motivation for multimodal fusion, Table 2 presents a comparison between ECG-only, PPG-only, and ECG-PPG fusion systems in terms of their signal characteristics, advantages, limitations, and typical application domains. As shown in the table, while ECG-only systems provide high temporal precision and reliable cardiac information, and PPG-only systems offer ease of use and low cost, both modalities have inherent limitations when used independently. Multimodal ECG-PPG fusion addresses these limitations by combining complementary electrical and hemodynamic information, thereby improving robustness and enabling advanced applications such as cuffless BP estimation, stress detection, and HRV analysis. As a result, multimodal fusion has demonstrated several advantages, including improved diagnostic accuracy, greater robustness to noise and missing data, and the ability to support continuous, wearable health monitoring [62,158,159,160].

**Figure 7:**
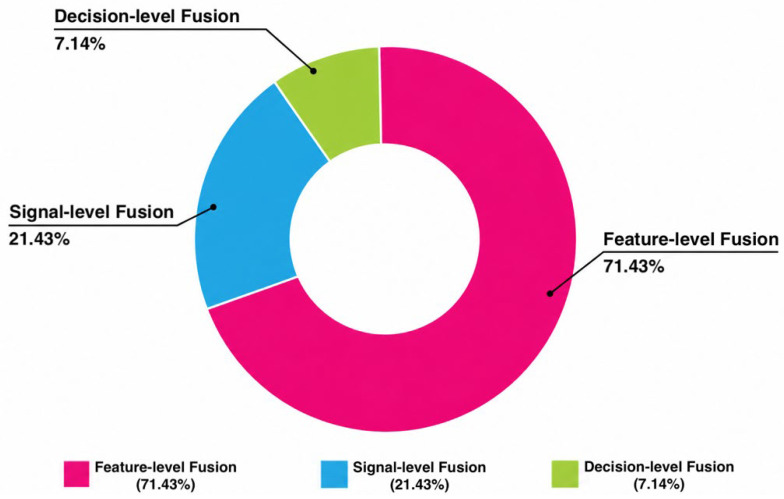
Distribution of fusion strategies in ECG-PPG multimodal systems, showing the dominance of feature-level fusion (71.43%) compared to signal-level (21.43%) and decision-level fusion (7.14%).

Feature-level fusion is the most common approach that combines ECG and PPG features into a single feature vector. HRV (ECG), PRV/morphology (PPG), and PTT/PAT (combined feature) are the common features that are used in stress monitoring and BP estimation [75,123]. Decision-level fusion (system-level integration) is also used in several studies that process ECG and PPG independently and combine outputs at the decision stage. In this method, sequential decisions (screening and confirmation), voting, or rule-based aggregation are the common strategies. It’s used for energy efficiency in wearable systems. The main limitation of this approach is that it does not exploit deep interaction between signals and lower information integration compared to feature-level fusion [62]. Signal-level fusion can be known as an emerging approach. This combines raw ECG and PPG signals using DL models and end-to-end learning. The multimodal system uses raw multi-channel signals with deep neural networks for HR estimation [119,125,161]. To provide a quantitative perspective on the prevalence of these fusion strategies, Figure 7 illustrates the distribution of fusion types used in the reviewed studies, highlighting the dominance of feature-level fusion compared to signal-level and decision-level approaches. It is important to note that ML- and DL-based approaches are not distinct fusion levels but rather modeling techniques applied within these fusion frameworks. Traditional ML methods typically rely on handcrafted features, whereas DL -based methods utilize architectures such as CNNs, RNNs/LSTMs, and hybrid models to automatically learn representations from multimodal data [122,156]. The following describes in detail how recent research has used multimodal fusion to obtain more complete, accurate, or useful results than those obtained from a single source, such as ECG or PPG, for applications such as cuffless BP estimation, stress monitoring, HR, and HRV monitoring.

### 5.2. Cuffless Blood Pressure Estimation Using ECG-PPG Multimodal Fusion

**Figure 8:**
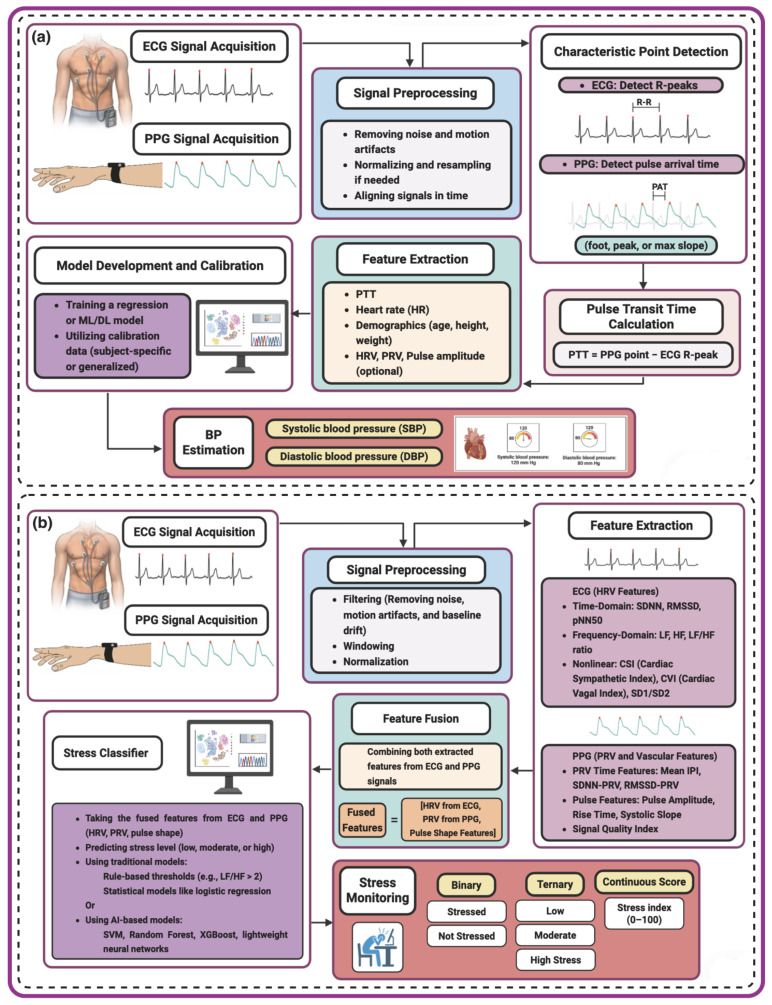
Overview of ECG-PPG multimodal fusion pipelines for physiological monitoring applications: (**a**) Cuffless blood pressure estimation, illustrating ECG and PPG signal acquisition, preprocessing, characteristic point detection, pulse transit time (PTT) calculation, feature extraction, model development and calibration, and systolic and diastolic blood pressure estimation; and (**b**) Stress monitoring, showing ECG and PPG signal acquisition, signal preprocessing, feature extraction from both modalities, feature fusion, stress classification, and stress level estimation, including binary, ternary, and continuous stress indices.

BP is simply the force exerted by circulating blood on the walls of the arteries and is measured in millimeters of mercury (mmHg) and is expressed as two numbers [33]. It is clinically defined as systolic blood pressure (SBP) and diastolic blood pressure (DBP) in a normal subject, and it is recorded as SBP/DBP [122]. SBP is defined as the maximum pressure in the arteries when the heart beats, that is, when blood is pumped out after the heart contracts. In a healthy adult, this value is typically around 110–130 mmHg [33,121,122]. DBP is defined as the minimum pressure in the arteries when the heart is at rest between refilling with blood after a heartbeat. DBP is defined as the minimum pressure in the arteries when the heart is at rest between refilling with blood after a heartbeat. For a healthy adult, that value is around 70–80 mmHg [33,121,122]. Figure 8a shows a summarized overview of a cuffless BP estimation pipeline designed based on ECG and PPG multimodal fusion, which researchers have used in recent studies.

Signal acquisition was the first step of the cuffless BP estimation pipeline. For this purpose, various hardware acquisition devices were designed to acquire synchronized PPG and ECG data. It is also noted that some studies have utilized curated, large datasets specifically developed for this purpose. Data from primary sources like MIMIC and VitalDB are particularly important among these. Filtering was used to minimize noise and motion artifacts and improve the signal-to-noise ratio, which was accomplished through preprocessing of the raw signal. The steps of feature point detection, PTT calculation, and feature extraction have been performed in various studies using different models. Numerous ML, DL, or regression models or algorithms have been utilized to estimate BP. Finally, the trained BP estimation model predicts SBP and DBP. Recent significant studies based on ECG and PPG multimodal fusion for cuffless BP estimation can be summarized in Table 3.

### 5.3. Mental Stress Detection Using ECG-PPG Multimodal Fusion

Mental health can be defined as a combination of emotional, social, and psychological well-being, and disorders of mental health can negatively affect a person’s thinking, mood, and behavior. Today, it is reported that more than 60% of the adult population suffers from stress due to various factors [17,75]. Stress is defined as a state of anxiety or mental tension caused by a difficult situation when events and responsibilities exceed one’s capacity. Stress can be divided into two types, which are physical stress caused by physical stimuli and mental stress caused by mental stimuli [128,129,171]. Stress has various adverse effects, including increased BP, atherosclerosis, and heart attacks [75]. The most fundamental step in managing stress is to correctly identify stress or assess its level. ECG and PPG multimodal fusion systems for the detection of mental stress have been proposed and developed recently. Figure 8b shows the summarized overview of the stress monitoring pipeline based on ECG and PPG multimodal fusion.

According to the stress monitoring pipeline, ECG and PPG signals are acquired initially. Filtering, windowing, and normalization are fulfilled in the preprocessing step. In the feature extraction, HRV features of the ECG signal, including time-domain, frequency-domain, nonlinear, and PRV features, as well as vascular features of the PPG signal, such as PRV metrics, pulse features, and signal quality index, are extracted. Extracted features from ECG and PPG are combined into a singular, cohesive feature vector in the feature fusion. In the stress classifier step, the stress level prediction is performed using fused features and traditional models (rule-based logic, logistic regression) or AI-based models (SVM, XGBoost, random forest, lightweight neural networks). The output, or predicted stress level, can be represented as a binary, ternary, or continuous score in Figure 8b, and it depends on the proposed system. Table 4 summarizes the most recent significant studies on multimodal ECG-PPG fusion for stress monitoring.

### 5.4. Heart Rate and Heart Rate Variability Monitoring Using ECG-PPG Multimodal Fusion

**Figure 9:**
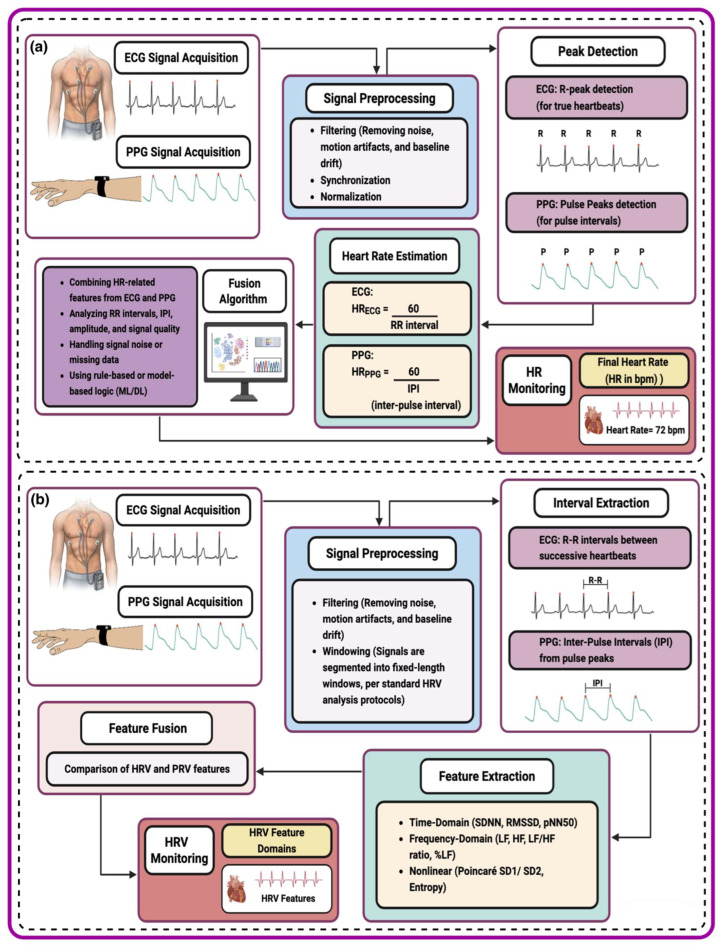
Overview of ECG-PPG multimodal fusion pipelines for cardiac monitoring applications: (**a**) Heart rate (HR) monitoring, illustrating ECG and PPG signal acquisition, preprocessing, peak detection, feature fusion, and final HR estimation; and (**b**) Heart rate variability (HRV) monitoring, showing signal acquisition, preprocessing, interval extraction, feature extraction and fusion, and HRV analysis across time-domain, frequency-domain, and nonlinear feature sets.

The number of heartbeats per minute (bpm) is considered HR, which can be expressed using ECG and PPG signals. HR can be calculated using the R-R interval in an ECG signal and the P-P interval in a PPG signal [124,172]. The variation in the time intervals between successive heartbeats is considered HRV [126]. Continuous long-term monitoring of HR is particularly valuable for applications in the medical field, such as circulatory, cardiovascular, and cerebrovascular management, as well as stress and emotional monitoring, and performance or fitness monitoring [124]. Through the use of nonlinear HRV analysis methods (such as DFA), more robust, accurate, and clinically relevant insights into cardiac rhythm and autonomic function can be obtained, especially in the detection and monitoring of arrhythmias [173,174]. Considering all these points, researchers have conducted several recent studies using ECG and PPG multimodal fusion, and a summarized overview of the HR and HRV monitoring pipeline can be seen in Figure 9a,b.

The first step of the HR monitoring pipeline based on ECG and PPG multimodal fusion is the acquisition of both signals separately. Then, signals are preprocessed, including filtering, synchronization, and normalization to ensure clear and aligned data before peak detection. In the peak detection step, some key points are detected, such as R-peaks in the ECG signal and pulse peaks in the PPG signal. HR estimation can be calculated using formulas that convert beat intervals into HR separately. In the fusion algorithm, both ECG and PPG information are combined to produce a reliable and fused HR estimation. Eventually, HR can be monitored in real time for clinical settings, fitness tracking, or any other applications. Similar to HR monitoring, ECG and PPG signals are detected in HRV monitoring. In the signal preprocessing, the signals are segmented into fixed-length windows, which is standard for HRV analysis. R-R intervals and inter-pulse intervals are detected in the interval extraction step. Standard HRV feature domains, including time domain, frequency domain, and nonlinear metrics, are extracted in the feature extraction of the HRV monitoring pipeline. Then, HRV and PRV features are compared and combined, and the final fused HRV results are obtained. A summary of the most recent significant studies based on ECG and PPG multimodal fusion for HR and HRV monitoring can be seen in Table 5.

## 6. Discussion

This comprehensive review presents a coverage of recent wearable monitoring technologies based on ECG and PPG and their modern multimodal fusion applications in the medical field. Recent studies demonstrate that combining ECG and PPG signals has become a very popular method today, and new wearable systems have been created through it. By combining the ECG signal, which provides information about the electrical activity of the heart, and the PPG signal, which provides information about vascular responses, with changes in blood volume, it has been possible to uncover more accurate and robust information.

The fundamental basis of all these designs is the theoretical background of ECG and PPG, and an understanding of the characteristics of each of these signals is essential, creating the necessary environment for integrating them into real-world applications. A comprehensive and detailed analysis based on recent literature has been presented on the main components of the ECG signal, namely the P wave, QRS complex, and T wave, as well as the time interval between different wave points and the J point, which are not addressed in many studies. Similarly, a comprehensive analysis has been provided based on the literature regarding the systolic peak, dicrotic notch, and diastolic peak, which are the components of the PPG signal. This review attempts to provide a basic understanding of the inherent characteristics of ECG and PPG, as well as the conditions that may arise from changes in those characteristics, before creating designs based on ECG and PPG multimodal fusion for applications in the medical field.

It is important to have an understanding of sensors when designing new wearable monitoring devices based on ECG and PPG. ECG-based wearable monitoring devices can be classified as single, dual, and multi-lead, and it has been observed that single-lead and dual-lead ECG sensors are widely used in most wearable devices. Probably due to their simplicity and low power consumption. The literature study revealed that sensors in PPG-based wearable monitoring devices can be divided into two types: transmissive and reflective, and that the sensors are selected based on the location of the wearable device. It was observed that wearable devices were designed to acquire ECG signals using ECG electrodes placed on the wrist, arm, or chest, and it is particularly notable that the majority of them were chest-based wearable devices. The authors have cited the main reasons for this as improved signal quality from the chest, reduced motion artifacts, and lead configuration. The placement of PPG-based devices spread across a wide range, with PPG-based devices with sensors placed on the wrist, finger, forehead, ear, and chest, and were seen to be used according to the need, convenience, and usability of the relevant study. Furthermore, this comprehensive review pays attention to the signal synchronization and preprocessing techniques for wearable PPG and ECG multimodal fusion systems. There is a thorough discussion of motion artifact removal, noise suppression, time alignment between ECG and PPG signals, analysis window size selection, and handling missing or corrupted data. These methods help researchers acquire high-quality signals throughout the development of ECG-PPG multimodal fusion systems.

In addition to developing wearable monitoring devices based on ECG or PPG, it was seen that multimodal fusion systems have been created by acquiring both ECG and PPG signals. The literature study indicates that researchers have focused on multimodal fusion systems with multiple sensors rather than relying on a single sensor source, such as ECG or PPG, due to their high accuracy, ability to compensate for missing or noisy data, and ability to continuously monitor for long periods. However, the researchers emphasize that when designing multimodal fusion systems, attention should also be paid to the practical issues inherent in multimodal fusion wearable devices, such as signal synchronization, power constraints, lack or scarcity of standardized datasets, and noise sensitivity.

This review contains a detailed analysis of several practical applications of multimodal systems in the medical field, based on research studies that have proven the most reliable and successful results experimentally and clinically, with literature evidence. The applications of ECG and PPG to real-time multimodal fusion studies, cuffless BP estimation, stress detection, HR, and HRV are analyzed here. Overviews of each application pipeline, based on ECG and PPG multimodal fusion, are presented in Figure 8 and Figure 9. Some of these steps vary depending on their respective applications. The steps of ECG and PPG signal acquisition, raw signal preprocessing, feature extraction, and model development were found to be used in many studies, with only minor differences depending on the applications. Several recent important studies based on ECG and PPG multimodal fusion for each application are summarized in Table 3, Table 4 and Table 5, thereby attempting to provide researchers with a better understanding by presenting a comprehensive analysis, including the results and evaluations of each system.

Most studies do not implement true ECG and PPG fusion. Instead, they rely on a single modality, either PPG or ECG alone. Even when fusion is used predominantly, feature-level fusion and limited exploration of hybrid or adaptive fusion frameworks. As an example, cooperative systems use decision-level fusion. For instance, they may employ PPG for initial screening followed by ECG for confirmation. There is a gap, as there is no standardized or optimal fusion strategy across applications. Signal synchronization is a critical bottleneck. ECG and PPG signals exhibit an inherent, dynamically varying physiological delay (PTT). It may be influenced by BP, vascular stiffness, and motion. Existing methods use R-peak alignment, cross-correlation, and timestamp synchronization. The main limitation of most studies is that they mostly assume static delay. There is also a lack of adaptive, real-time synchronization models in current research.

It has been observed that various wearable devices have been used to acquire ECG and PPG signals for ECG and PPG multimodal fusion applications, and it is unique that devices have been developed to acquire both signals from a single location in some studies. For example, attempts to capture both signals from the finger, wrist, ear, hand, and chest have been successful, taking the detection of physiological signals in a new direction. However, another important point revealed by this review is that since very few have been used for practical applications by receiving signals from the same location, there is still much room for new studies for researchers interested in this topic. In addition, it was also revealed that the Biopac, MIMIC-I, MIMIC-II, MIMIC-III, VitalDB, and MIT-BIH datasets were used in the development of ECG and PPG multimodal fusion systems, almost all of which are AI-based research. Some studies created a dataset using only data from wearable devices that they developed that could detect ECG and PPG signals, while other researchers used data from wearable devices they developed for their studies, as well as the aforementioned dataset for training and testing their systems, trying to obtain more accurate and reliable results. As mentioned earlier, it is also important to highlight through this review that some studies used only previously available standard datasets (Biopac, MIMIC-I, MIMIC-II, MIMIC-III, VitalDB, and MIT-BIH).

The authors highlight through the literature on previously developed ECG and PPG monitoring wearable multimodal fusion application systems that the outcome of these systems varies due to various interrelated factors. Sensor performance and signal quality are strongly affected by factors such as the way the electrodes are placed on the skin, motion artifacts, the skin tone and thickness of the person using the device, and the nature of the signal synchronization. Moreover, it is emphasized that the final result can be influenced not only by the sample ratio used and the wearable position of each device but also by physiological characteristics such as BMI, sex, and age. The accuracy, reliability, and quality of the outputs of ECG and PPG fusion systems vary depending on the fusion method used, the complexity of the model, the size of the dataset used, and the type of features used in the systems. Researchers should pay greater attention to these issues when designing wearable multimodal fusion systems based on ECG and PPG, as well as on other physiological signals, to improve their quality and reliability. Future research in ECG-PPG multimodal fusion should focus on developing AI-assisted, adaptive, and real-time fusion frameworks that can operate efficiently on wearable edge devices.

## 7. Conclusions

This review presents a broad coverage of recent wearable physiological monitoring technologies based on ECG and PPG and their modern multimodal fusion applications in the medical field, namely cuffless BP estimation, stress monitoring, HR, and HRV monitoring. Studies of these applications clearly demonstrate that full ECG-PPG multimodal fusion systems achieve significantly better performance than systems based on ECG or PPG alone. In addition, this provides a detailed description of the basic theoretical background with the characteristics of the ECG and PPG signals required to develop wearable multimodal fusion applications based on ECG and PPG, as well as the recently used ECG and PPG wearable monitoring techniques. This extensively reviewed the pros and cons of using multimodal fusion, including improving diagnostic accuracy, rather than drawing conclusions using only an ECG or PPG signal. Also, a comprehensive and comparative analysis of studies with modern multimodal fusion applications in the aforementioned medical field is presented based on the literature. This review discusses the key limitations and challenges of ECG-PPG wearable multimodal fusion systems. Considering these aspects, this review seeks to provide valuable guidance for researchers developing wearable multimodal fusion applications based on ECG and PPG.

## Figures and Tables

**Table 1:** Overview of synchronization and preprocessing techniques and their applications in ECG-PPG multimodal.

Category	Method Type	Purpose/Function	Typical Application	Usage Trend
Synchronization	PTT-based alignment/cross-correlation	Align ECG-PPG signals using physiological delay	Cuffless BP estimation (PAT/PTT), HRV analysis	Most commonly used
	Timestamping/resampling	Align signals from different sampling rates/devices	Wearable multi-device systems, continuous monitoring	Commonly used
	DTW/phase-based/ML-based alignment	Handle nonlinear delay and temporal variability	Robust real-world monitoring, stress detection systems	Less common; emerging
Artifact Removal	Bandpass/adaptive filtering	Remove motion-related noise	General wearable monitoring (HR, BP, stress)	Most commonly used
	SQI/IMU-assisted correction	Detect and reduce motion artifacts	Ambulatory monitoring, fitness tracking	Commonly used
	ICA/PCA/BSS	Separate physiological signal from noise sources	Multichannel ECG-PPG systems, research-grade setups	Less common
	ML-based artifact suppression	Learn and suppress complex artifacts	Dynamic environments, stress and BP estimation systems	Increasingly used
Noise Reduction	Bandpass/wavelet denoising	Remove noise while preserving signal morphology	ECG/PPG preprocessing for all applications	Most commonly used
	Baseline correction/detrending	Remove low-frequency drift	Long-term monitoring, HRV analysis	Commonly used
	Savitzky–Golay/nonlinear filtering	Smooth signals without distorting features	Feature extraction (PPG morphology, HR estimation)	Less common
	EMD/VMD/SSA	Decompose signals for adaptive denoising	Non-stationary signals in BP and stress analysis	Emerging
	DL denoising	Learn noise patterns from data	Advanced BP estimation and multimodal fusion systems	Increasingly used
Missing Data Handling	Interpolation/reconstruction	Recover missing segments	Continuous wearable monitoring (HR, BP tracking)	Commonly used
	Segment rejection/weighting	Ignore low-quality segments	Real-time monitoring systems	Commonly used
	Modality-aware fusion/redundancy	Adapt when one signal fails	Multimodal ECG-PPG fusion systems	Emerging
Analysis Window Selection	Short (2–10 s)	Low-latency processing	HR estimation, arrhythmia detection	Common
	Medium (10–30 s)	Stable feature extraction	Cuffless BP estimation (PAT/PTT), multimodal fusion	Common
	Long (30 s–5 min)	Capture autonomic trends	Stress detection, HRV monitoring	Common

**Table 2:** Comparison of ECG-only, PPG-only, and ECG-PPG multimodal fusion systems.

System Type	Signal Characteristics	Advantages	Limitations	Typical Applications
ECG-only	Electrical cardiac activity with high temporal precision	High accuracy in cardiac event detection, Precise R-peak timing, Reliable for rhythm analysis	Requires electrodes and proper skin contact, Less comfortable for long-term wear, Limited peripheral information	Arrhythmia detection, HR, HRV, Stress analysis [30,31,32,167,168]
PPG-only	Optical measurement of blood volume changes in peripheral vessels	Easy to wear, Low cost, Suitable for continuous and consumer-grade monitoring	Highly sensitive to motion artifacts, ambient light, and perfusion variability, Indirect cardiac measurement	HR, blood oxygen saturation, BP estimation, Stress detection, Sleep monitoring, Atrial fibrillation detection, Basic physiological monitoring [39,43,97,108,169,170]
ECG-PPG Fusion	Combined electrical and hemodynamic information	Improved accuracy and robustness, Complementary information enables advanced physiological analysis	Increased system complexity, Higher computational and power requirements, Synchronization challenges	Cuffless BP estimation (PAT/PTT), stress detection, HRV, multimodal health monitoring, Arrhythmia detection [17,62,75,119,122,123,124,125,126,140,153,154,156,157,158,160,161,162,163,164,165,166]

**Table 3:** Recent significant studies based on ECG and PPG multimodal fusion for cuffless blood pressure estimation.

Study	Data and Setup	Fusion and Model	Features and Synchronization	Performance and Validation	Strengths and Limitations
Wanda et al. (2024) [162].	Chest ECG and finger PPG; MIMIC II (100 recordings, 125 Hz) and real subjects	Feature-level fusion and ANN (3-layer SBP and 4-layer DBP)	PTT, ECG (HR), and PPG morphological features (16); PTT-based synchronization (R-peak to PPG peak)	SBP MAE ± 13.89 mmHg and DBP MAE ± 5.5 mmHg; Dataset and real-time validation	Low cost, embedded-ready, continuous monitoring; High SBP error, motion sensitivity, limited metadata, calibration required
Shirong Qiu et al. (2023) [123].	ECG and PPG wearable system; Biopac (short-term), MIMIC-I (~20 h), JOCOC (36-day elderly)	Feature-level fusion (Pattern Fusion) and multi-module regression model	Ts/Tc, Tc, PTT, SV, HR, and multimodal features; ECG-PPG alignment via R-peak (PTT and PAT)	SBP MAE 3.65–6.84 mmHg and DBP MAE 3.65–4.56 mmHg; Multi-scenario validation (short, ICU, long-term)	High accuracy, scenario-adaptive, interpretable and robust; Requires calibration, complex model, signal quality dependent
Li et al. (2022) [163].	ECG handheld device and finger PPG; 85 subjects (AAMI) and outpatient data	Feature-level fusion and residual DNN with hierarchical regression	PPG morphology, FDPPG, SDPPG, WPD, ECG-PPG fiducial features, entropy and demographics (~4900); Fiducial-based synchronization (PAT-like)	SBP MAE 7.55 mmHg and DBP MAE 5.96 mmHg; AAMI protocol, subject-split, calibration-free	AAMI evaluation, calibration-free, large feature set, robust preprocessing and good generalization; High feature complexity, moderate SBP error, complex pipeline
Zainab Jamil et al. (2025) [119].	Clinical ECG and fingertip PPG; PulseDB (5361 subjects, MIMIC-III and VitalDB)	Signal-level fusion and self-attention ResUNet	Deep features using ResUNet and attention; Implicit synchronization (aligned 10 s windows)	Calibration-free: SBP 8.11 mmHg and DBP 5.12 mmHg; Calibration-based: SBP 4.45 mmHg and DBP 1.13 mmHg; Cross-dataset and AAMI/IEEE/BHS validation	Large dataset, strong generalization, fairness analysis, attention improves performance; Weaker SBP, no wearable validation, performance drop in calibration-free mode, high complexity
Shaikh et al. (2025) [122].	ECG and PPG devices; PulseDB (3027 subjects, MIMIC-III and VitalDB)	Feature-level fusion with dual-stream CNN and Bi-LSTM	Deep features; Explicit synchronization using aligned 10 s segments (125 Hz)	SBP MAE 5.16 mmHg and DBP MAE 3.24 mmHg; Fivefold cross-validation, hold-out testing, AAMI/IEEE/BHS	Strong performance, improved feature extraction, large dataset and demographic analysis; Slight SBP drop, data imbalance, ICU-only data, no wearable validation
Rebecca Mieloszyk et al. (2022) [154].	Wrist ECG (tonometry) and PPG; 1125 participants (ambulatory and lab, 24 h monitoring)	Feature-level fusion and ridge regression	rPAT, PTT, waveform morphology, HR and time-of-day; Explicit synchronization using QRS cross-correlation	SBP ~0.86 ± 8.7 mmHg and DBP ~0.75 ± 5.9 mmHg; Stratified cross-validation, bootstrap and baseline comparison	Large real-world dataset, ambulatory evaluation, multi-sensor comparison, diverse cohort; Signal quality issues, discomfort, missing data, calibration dependency, weaker performance in elderly
Aayushman Ghosh et al. (2023) [164].	Clinical ECG and PPG (ICU); MIMIC-II (90 patients)	Feature-level fusion and boosting models (CatBoost and AdaBoost)	Bio-inspired ECG and PPG morphological features (16); implicit synchronization (same system)	SBP MAE 3.81 mmHg and DBP MAE 2.22 mmHg (r ≈ 0.90/0.83); 10-fold cross-validation and Bayesian optimization	Interpretable features, strong performance (AAMI and BHS Grade A), low computational cost; Small dataset, ICU-only, limited generalization

**Table 4:** Recent significant studies based on ECG and PPG multimodal fusion for stress monitoring.

Study	Data and Setup	Fusion and Model	Features and Synchronization	Performance and Validation	Strengths and Limitations
Chongyan Chen et al. (2019) [156].	Chest ECG and wrist PPG; 6 subjects, lab stress task (100 Hz)	No direct ECG and PPG fusion and Random Forest (best, compared with SVM, NB and MLP)	HRV features from RR and PP intervals (time, frequency and nonlinear); Simultaneous recording and interval filtering without explicit fusion alignment	Accuracy: ECG 97.58–97.94% and PPG 98.00–98.48%; Generalization F1: ECG 0.797 and PPG 0.802–0.807; 10-fold CV and leave-one-subject-out	Shows wrist PPG HRV comparable to ECG for stress detection; Very small sample, lab-only setting, limited generalization
Glenn Fernandes et al. (2024) [75].	Flexible chest ECG and PPG patch; 11 subjects, lab stress (~1005 min data)	Feature-level fusion and Gradient Boosting Machine	HRV (ECG), PPG morphology, statistical features and PAT; Explicit synchronization via ECG R-peak to PPG fiducials	F1: 85.5% (perceived stress) and 87.7% (physiological stress); Stratified 5-fold CV	True multimodal fusion, wearable comfort, interpretable model and PAT importance; Small dataset, lab-induced stress, limited real-world validation
Mouna Benchekroun et al. (2022) [165].	Chest ECG and earlobe PPG; 46 subjects, lab stress protocol (~1000 windows)	No direct ECG and PPG fusion, separate modality modeling and Random Forest (optimized)	HRV features (time, frequency and nonlinear); Simultaneous recording but separate processing	ECG: F1 0.83 and AUC 0.91; PPG: F1 0.82 and AUC 0.92; 10-fold CV and cross-sensor validation	Shows PPG as surrogate for ECG in stress detection; Lab-controlled, HRV-only features, performance drop in cross-sensor testing
Anuja Pinge et al. (2022) [157].	Chest ECG and wrist PPG smartwatch; 5 subjects, multi-stressor (~13k samples)	No ECG and PPG fusion, device-wise independent modeling and comparison and Random Forest	HRV and statistical features from HR and RR intervals; Implicit synchronization via time-aligned streams	F1: 0.85 (ECG) and 0.80–0.82 (PPG); HR RMSE: 5.2–10.23 bpm; Leave-one-person-out CV	Demonstrates smartwatch PPG comparable to ECG; Very small dataset, no fusion, lab-only, limited generalization
Lili Zhu et al. (2022) [17]	Wrist PPG (Empatica E4) and chest ECG (RespiBAN); WESAD (15) and CLAS (62)	Feature-level fusion and stacking ensemble learning	ECG, PPG and EDA features (HRV, morphology, statistical); Implicit synchronization with 30 s windows	Best accuracy: 86.4% (EDA), fusion ≈ 67.7–67.8%; Evaluated on two datasets	Comprehensive multimodal analysis and benchmark datasets; Fusion underperforms single modality, feature redundancy, limited real-time validation

**Table 5:** Recent significant studies based on ECG and PPG multimodal fusion for heart rate and heart rate variability monitoring.

Study	Data and Setup	Fusion and Model	Features and Synchronization	Performance and Validation	Strengths and Limitations
Yongbin Lee et al. (2025) [62].	Wrist wearable with ECG, multi-channel PPG and IMU; Multiple datasets (BAMI, ISPC, SBH clinical, MIMIC PERform AF) and pilot clinical study	Decision-level cooperative fusion and AF detection pipeline (FSM and entropy-based methods)	PPG features (HR, pseudo-PPI, variability and spectral), ECG RR intervals and IMU motion features; Synchronized acquisition via integrated wearable system	HR MAE ≈ 1.16–1.31 bpm and AF detection accuracy 0.9847 (clinical); Multi-dataset validation and clinical pilot	True multimodal system, real wearable implementation, robust motion artifact removal, adaptive fusion strategy; Complex system, ECG not continuous, user-dependent ECG triggering
Xinxia Li et al. (2022) [153].	Upper-arm PPG wearable and chest ECG; 22 subjects, real ICU work conditions (day and night shifts)	No ECG and PPG fusion, concurrent comparative monitoring and statistical correlation analysis	HR and HRV features (LF, HF, LF/HF and percent LF); Simultaneous recording with interval-based averaging	HR correlation r = 0.974 and HRV moderate agreement; Bland–Altman and cross-condition validation	Real working-condition study, strong HR agreement, practical wearable applicability; Not a fusion model, small sample, limited duration and moderate HRV agreement
Philip Mehrgardt et al. (2021) [125].	Finger-worn multimodal PPG device and chest ECG; 21 subjects with activity data (stationary, walking, running)	Signal-level fusion and deep neural network regression	Raw multivariate signals (PPG, pressure and IMU) with ECG reference; precise alignment using ECG R-peaks and window segmentation	HR AAE < 1 bpm and RMSE ≈ 28–40 ms; Leave-one-out cross-validation	High accuracy across motion conditions, true multimodal fusion, improved performance using pressure sensing; Small dataset, ECG used only as reference, limited real-world variability
Haozhe Tian et al. (2023) [126].	Ear-worn ECG and PPG system; 7 subjects, multi-session recordings (breathing and mental tasks)	Feature-level fusion and machine learning classification (RF, SVM, NB)	ECG HRV features and PPG breathing features; Synchronized acquisition with reference ECG alignment	R-peak detection F1 up to 0.97 and classification accuracy up to 95%; Cross-subject validation	Comfortable ear-based sensing, improved classification with multimodal features, robust R-peak detection; Small dataset, controlled scenarios, limited generalization
Amr Farhan et al. (2024) [166].	Chest ECG and finger PPG; 53 cardiac patients from PhysioNet BIDMC dataset	No ECG and PPG fusion, Correlation and agreement analysis (HRV vs. PRV) and statistical agreement analysis	HRV and PRV features (time and frequency domain); Synchronized dataset with independent extraction	Very high correlation (r ≈ 0.99–1.0) and strong agreement; Statistical validation	Strong evidence of PRV as surrogate for HRV, large clinical dataset; Not a fusion study, no predictive model, offline analysis only
Qingxue Zhang et al. (2017) [124].	Ear-worn ECG and PPG wearable; 14 subjects with motion scenarios	Feature-level fusion and machine learning (SVM, DTW, clustering and regression)	PTT, HR and ECG features; explicit synchronization using ECG R-peak and PPG foot	HR error 0.8 ± 2.7 bpm and BP error −1.4 ± 5.2 mmHg; Train-test validation	True multimodal fusion, robust to motion, wearable design; Small dataset, complex pipeline, requires calibration
M. G. Srinivasa et al. (2016) [161].	Chest-worn wearable belt with ECG and PPG; small-scale continuous monitoring (~4 h)	Signal-level fusion and embedded signal processing system	ECG, PPG, HR, PTT and temperature; Implicit synchronization via QRS and PPG peaks	Qualitative validation (comparable to standard devices); Real-time monitoring	Complete wearable prototype, multi-parameter monitoring, real-time transmission; No ML methods, limited validation, small dataset

## Data Availability

The data presented in this study are openly available in the articles cited throughout the manuscript. No new datasets were generated during this study.

## Abbreviations

The following abbreviations are used in this manuscript:

ECG	Electrocardiogram
EKG	Electrocardiogram (alternative term)
PPG	Photoplethysmography
EMG	Electromyography
EOG	Electrooculography
EEG	Electroencephalogram
SCG	Seismocardiography
HR	Heart Rate
HRV	Heart Rate Variability
BP	Blood Pressure
SBP	Systolic Blood Pressure
DBP	Diastolic Blood Pressure
SpO_2_	Blood Oxygen Saturation
AF	Atrial Fibrillation
AHI	Apnea–Hypopnea Index
RR	R–R Interval
PR	P–R Interval
QT	Q–T Interval
ST	S–T Segment
PTT	Pulse Transit Time
PAT	Pulse Arrival Time
PPI	Peak-to-Peak Interval
ΔT	Time Difference
DWT	Discrete Wavelet Transform
CWT	Continuous Wavelet Transform
MODWT	Maximal Overlap Discrete Wavelet Transform
EMD	Empirical Mode Decomposition
VMD	Variational Mode Decomposition
SSA	Singular Spectrum Analysis
FFT	Fast Fourier Transform
ML	Machine Learning
DL	Deep Learning
ANN	Artificial Neural Network
CNN	Convolutional Neural Network
RNN	Recurrent Neural Network
LSTM	Long Short-Term Memory
SVM	Support Vector Machine
RF	Random Forest
IMU	Inertial Measurement Unit
LED	Light Emitting Diode
IoT	Internet of Things
MCT	Mobile Cardiac Telemetry
ILR	Implantable Loop Recorder
BioZ	Bioimpedance
GSR	Galvanic Skin Response
EDA	Electrodermal Activity
SKT	Skin Temperature
MAE	Mean Absolute Error
RMSE	Root Mean Square Error
CV	Cross-Validation
LF	Low Frequency
HF	High Frequency
LF/HF	Low Frequency to High Frequency Ratio
EWS	Early Warning Score
VR	Virtual Reality
